# Deregulation of PRDM5 promotes cell proliferation by regulating JAK/STAT signaling pathway through SOCS1 in human lung adenocarcinoma

**DOI:** 10.1002/cam4.5251

**Published:** 2022-09-20

**Authors:** Yuanyuan Ren, Ye Wang, Lijiao Fang, Mengchu Ma, Lin Ge, Chao Su, Lingbiao Xin, Jinyan He, Jie Yang, Xin Liu

**Affiliations:** ^1^ Key Laboratory of Immune Microenvironment and Disease (Ministry of Education) Department of Biochemistry and Molecular Biology School of Basic Medical Sciences Tianjin Medical University Tianjin China; ^2^ Sinovac Biotech Co. Ltd Beijing China; ^3^ Qilu Medical University Shandong China; ^4^ University of Hong Kong‐Shenzhen Hospital Shenzhen China

**Keywords:** JAK/STAT signaling pathway, lung adenocarcinoma, PRDM5, SOCS1

## Abstract

**Background:**

PRDM5 is considered a tumor suppressor in several types of solid tumors and is involved in multiple cellular processes. However, target genes regulated by PRDM5 in lung cancer and its potential mechanism are poorly defined.

**Methods:**

Survival analysis was conducted using Kaplan‐Meier estimates based on the online databases. RNA‐sequencing and bioinformatics analysis were performed to identify the differentially expressed genes in PRDM5‐overexpressed A549 cells.

**Results:**

We observed deregulated PRDM5 in several lung adenocarcinoma cell lines and its association with a poor prognosis. PRDM5 overexpression inhibited the proliferation of lung adenocarcinoma cells in vitro and suppressed tumor growth in a xenograft model. PRDM5 upregulated the promoter activity of SOCS1, which then inhibited the phosphorylation of JAK2 and STAT3.

**Conclusions:**

Our study suggests that the low expression of PRDM5 promotes the proliferation of lung adenocarcinoma cells by downregulating SOCS1 and then upregulating the JAK2/STAT3 signaling pathway.

## Graphical abstract

Our study suggests that deregulation of PRDM5 promotes the proliferation of lung adenocarcinoma cells by downregulating SOCS1 and then upregulating the JAK2/STAT3 signaling pathway. The findings will provide significant clues and evidence for targeted treatment of lung adenocarcinoma in the clinic.

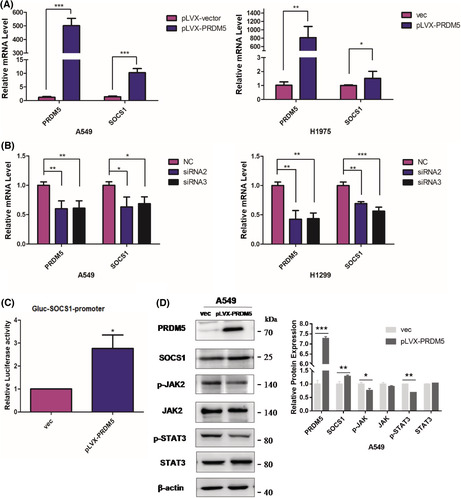

## 1 INTRODUCTION

Lung cancer is one of the most malignant tumors worldwide due to its poor prognosis and lack of an effective diagnostic strategy. According to the data from the International Agency for Research on Cancer 2018,^1^ lung adenocarcinoma (LUAD), which accounts for approximately 40% of non‐small cell lung cancer (NSCLC), has been the top lung cancer subtype in terms of incidence. Although various strategies have been developed in LUAD treatment,^2^ resistance of LUAD to targeted therapies remains a major challenge for treatment effectiveness.^3^, ^4^ Hence, more considerable effort on exploring the new regulatory mechanisms of lung carcinogenesis will be crucial to the diagnosis and targeting therapy for LUAD.

The PRDM (PRD‐BF1 and RIZ homology domain containing) proteins constitute a family of transcriptional regulators characterized by a PR domain in the N‐terminus and a number of C2H2 zinc fingers near the C‐terminus.^5^ Within recent years several PRDM members have been delineated associated with pathological conditions, particularly in cancers. Tumor suppressor activity in various solid tumors has also been found for PRDMs such as PRDM1‐3, PRDM5, and PRDM12.^6^ Recently, several studies have revealed the role of PRDM5 in different types of cancer, demonstrating that it functions as a tumor suppressor to regulate its target genes at the transcriptional level or by interacting with epigenetic regulators.^7^, ^8^, ^9^, ^10^, ^11^ Although a multitude of genes modulated by PRDM5 have been reported, such as p53, MYC, and MDM2,^10^, ^12^ the exact role of PRDM5 targets in lung carcinogenesis and its relative mechanisms are rarely characterized. In addition to the findings of deregulation of PRDM5 in numbers of solid tumor types,^8^, ^13^, ^14^ our study also showed that PRDM5 acted as a tumor suppressor in LUAD. More important, we suggested that SOCS1 might be one of the target genes of PRDM5 and mediated the proliferation of LUAD cells by JAK2/STAT3 signaling pathway.

In our study, we examined the downregulation of PRDM5 expression and its association with a poorer survival rate in LUAD patients. Similarly, knockdown of PRDM5 promoted cancer cell growth in an in vitro assay. Furthermore, in gain‐of‐function experiments, PRDM5 overexpression suppressed cellular growth both in vitro and in vivo. According to our RNA‐sequencing and KEGG pathway analysis, we determined that increased expression of PRDM5 upregulated SOCS1 expression and inhibited the proliferation of LUAD cells by suppressing the JAK2/STAT3 signaling pathway. Our findings might provide significant clues and evidence for the targeted treatment of lung adenocarcinoma in the clinic.

## 2 MATERIALS AND METHODS

### 2.1 Cell culture

Epithelial BEAS‐2B cells, which were isolated from normal human bronchial epithelium, and A549, H358, H520, H1299, and H1975 cells, which are lung adenocarcinoma cell lines, were all provided by Dr. Zhenyi Ma (Tianjin Medical University). In addition, 293 T cells were acquired from the American Type Culture Collection (ATCC). Dulbecco's modified Eagle medium (DMEM; BI) was used to culture 293 T cells and BEAS‐2B cells. Roswell Park Memorial Institute (RPMI)‐1640 medium was used to culture the H1975, H1299, H358, H520, and A549 cell lines. In addition, all media were supplemented with 10% FBS (BI), and a 1% penicillin/streptomycin solution was added to the culture base if needed.

### 2.2 Construction of plasmids and stable cell lines

Total RNA was extracted and transcribed into cDNA for the construction of recombinant plasmid pLVX‐IRES‐Puro‐PRDM5‐Flag (pLVX‐IRES‐Puro vector, 632,183, Clontech Laboratories). The two types of envelope expression plasmids (pMD2.G and psPAX2) and the constructed pLVX‐IRES‐Puro‐PRDM5‐Flag were then cotransfected into 293 T cells by PEI (Santa Cruz, sc‐360988A, 1 mg/ml) (pMD2.G:psPAX2:pLVX‐PRDM5/VEC = 3.95:7.3:11.25). Supernatants containing lentivirus were collected, centrifuged, and filtered after 24 h and 48 h of transfection. A549 and H1975 cells were subjected to lentivirus infection for 24 h. Stable cell lines overexpressing PRDM5 were obtained by treating the cells with 2 μg/ml puromycin for 3 days. The full‐length SOCS1 promoter was cloned into GLuc‐ON™ promoter reporter clones (pEZX‐PG04, GeneCopoeia) to construct luciferase reporter plasmids.

### 2.3 RNA interference (RNAi)

siRNA‐NC and si‐PRDM5 were purchased from GenePharma. The sequences of PRDM5 siRNA were as follows: siRNA2: 5′‐GCUUGUCCUCAAUGUGAAUTT‐3′; siRNA3: 5′‐GGUGUGAGCUAUGUAAUAATT‐3′. siRNA was dissolved in DEPC water (100 μM) and then transfected into cells with Lipofectamine TM 2000 (11,668,500, Invitrogen) according to the manufacturer's instructions.

### 2.4 Western blotting and antibodies

Protein samples were extracted from different groups of cells, and the protein concentration was determined by the BCA (Thermo Fisher Scientific) method. The primary antibodies used were as follows: anti‐PRDM5 (sc‐376,277, Santa Cruz), anti‐SOCS1 (3950 T, CST), anti‐STAT3 (4904, CST), anti‐pSTAT3 (Tyr 705) (3950 T, CST), anti‐JAK2 (17670‐1‐AP, Proteintech), anti‐pJAK2 (Y1007) (YP0155, ImmunoWay), and anti‐β‐actin (A1978; Sigma). Anti‐rabbit IgG (H + L), HRP conjugate (Promega, W4011), and HRP‐anti‐mouse IgG antibody (Cell Signaling Technology, 7076S) were used as secondary antibodies for the abovementioned blots. Image J 2X software (NIMH) was used to digitize the band density.

### 2.5 Gene expression analysis

Total RNA (1000 ng) was used in the reverse transcription step to prepare the cDNA. Quantitative RT‐PCR was performed on an ABI‐Step One Plus (Thermo) using SYBR Green (4,913,850,001, Roche). Specific primer pairs for PRDM5 were as follows: forward 5′‐ACTCGATGCTGAACTGAAGGA‐3′ and reverse 5′‐GTTCTTCAGTGCACAGCGAAA‐3′. The relative expression levels of RNA were calculated by the 2^−ΔΔCt^ method. Each sample was tested in triplicate.

### 2.6 Cellular proliferation assays

A total of 1 × 10^3^ A549 cells were seeded into 96‐well culture plates to overexpress or knockdown PRDM5. Cell growth was tested every day according to the instructions of the MTT kit (CyQUANT™, Invitrogen™) for 6 days of culturing. The absorbance value at 490 nm was determined by a microplate reader (Varioskan Flash, Thermo).

### 2.7 Colony formation assay

Lung adenocarcinoma cells with PRDM5 overexpression or knockdown were seeded into 6‐cm plates with 2 × 10^3^ cells in each plate. After approximately 7–10 days in culture, macroscopic single colonies of cells were fixed with 4% paraformaldehyde. The fixed cells were then stained with crystal violet, and the number of colonies that contained more than 50 cells was counted by ImageJ software.

### 2.8 Dual‐Luciferase Reporter assay

The GLuc‐SOCS1 promoter (full‐length) (0.5 μg/50 μl) and PRDM5‐Flag (0.5 μg/50 μl) plasmids were cotransfected into cells by PEI, and the supernatant was collected for assessment within 24–48 h after transfection. The supernatant was assayed for secreted alkaline phosphatase (SEAP) and Gaussian luciferase (GLuc) according to the instructions of the detection kit (SPDA‐D010, GeneCopoeia). The experimental results were obtained from the ratio of GLuc/SEAP. All samples were detected by a Glo Max™ 96 Microplate Luminometer (Promega).

### 2.9 Animal experiments

All animals were provided 12 h of light and 12 h of darkness and had free access to water and ordinary feed (Beijing KeAoXieLi Feed Limited Company). Each female Balb/C nude mouse was subcutaneously injected with an A549 cell suspension containing 1 × 10^6^ cells, which was diluted with 0.2 ml PBS. Nude mice were divided into an experimental group (pLVX‐IRES‐PRDM5, *n* = 5) and a control group (empty vector, *n* = 5) according to the different types of cells injected. After tumor formation, the long and short diameters of the tumor were monitored daily and recorded. All animal experiments were approved by the committee on the use and care of animals of Tianjin Medical University.

### 2.10 Bioinformatic analyses

#### 2.10.1 RNA‐sequencing

pLVX‐IRES‐PRDM5 or a vector‐control plasmid was transiently transfected into A549 cells with Lipofectamine TM 2000 (11,668,500, Invitrogen) according to the manufacturer's instructions. After culturing for 48 h, total RNA samples were extracted from cells via TRIzol reagent (Invitrogen) and used to construct RNA sequencing libraries (samples from three separate experiments). Sequencing and bioinformatics analysis of the library was performed by BGI Genomics (BGI Genomics Co., Ltd.). The differentially expressed genes of the two groups were quantitatively analyzed after standardization (*p* < 0.05, fold change = 1.5). Then, cluster analysis, volcano plot, GO function, and KEGG pathway analysis were performed on the identified genes.

Kaplan–Meier survival analysis was used to evaluate the association between the survival rate of patients and the PRDM5 expression levels based on the online databases.

### 2.11 Flow cytometry analysis

Lung adenocarcinoma cells with overexpressed PRDM5 or vector control were harvested and adjusted the cell density to 1–5 × 10^6^ cells/ml. The cells were stained for cell cycle detection according to the manufacturer's instructions (Cell Cycle Assay Kit‐PI/RNase, C543, DOJINDO). The cell cycle distribution was analyzed by a flow cytometry (BD Biosciences FACSVerse, Becton Dickinson).

### 2.12 Statistical analysis

IBM SPSS Statistics 20 software was selected for data processing, and the results of the biological triplicate experiments are presented as the mean ± SD. According to the results of statistical analysis, a *p* value<0.05 was considered statistically significant.

## 3 RESULTS

### 3.1 Expression of PRDM5 was frequently reduced in lung adenocarcinoma and was related to a poor prognosis

**FIGURE 1:**
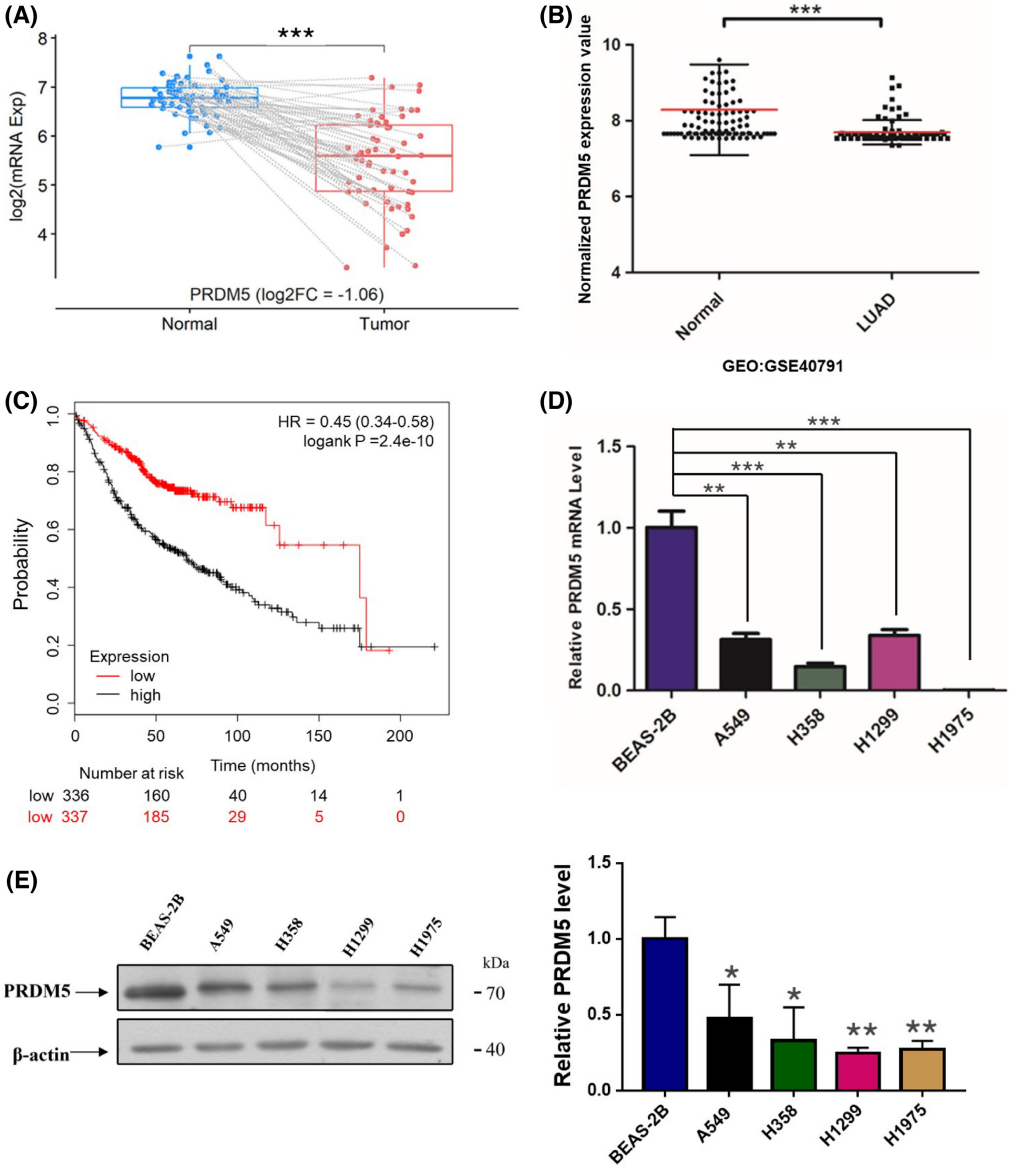
PRDM5 is frequently reduced in lung adenocarcinoma and is related to a poor prognosis. (A) PRDM5 mRNA expression in tumor‐normal paired lung adenocarcinoma samples (*n* = 58). (B) RNA‐sequencing profiles of PRDM5 (GEO: GSE40791) in human normal lung tissues (*n* = 100) and LUAD (*n* = 94). (C) Kaplan–Meier survival analysis. The mRNA level (D) and protein expression (E) of PRDM5 were examined in lung cell lines (*n* = 5). *p < 0.05; **p < 0.01; ***p < 0.001.

High‐throughput screening of 58 tumor‐normal paired lung adenocarcinoma (LUAD) samples revealed that PRDM5 expression in adenocarcinoma tissues was lower than that in para‐carcinoma tissues (Figure 1A). To identify the PRDM5 expression profiles in lung adenocarcinoma, we examined the expression status of PRDM5 in several lung adenocarcinoma cell lines and immortalized human bronchial epithelial BEAS‐2B cells. Reduced PRDM5 expression was observed in lung adenocarcinoma cells when compared with BEAS‐2B cells (Figure 1D,E). After analyzing the data of PRDM5 expression in human lung samples (GEO: GSE40791), we observed decreased mRNA levels of PRDM5 in lung adenocarcinoma tissues compared with normal lung tissues (Figure 1B), which was in accord with the results from the BEAS‐2B cells and lung adenocarcinoma cell lines. Consistent with the results above, Kaplan–Meier survival analysis showed that lower PRDM5 expression was associated with a poorer survival rate in lung adenocarcinoma patients (Figure 1C). These data indicated that PRDM5 expression decreased in lung adenocarcinoma cell lines and that deregulated PRDM5 predicted poor survival of patients.

### 3.2 Upregulation of PRDM5 inhibited lung adenocarcinoma cell proliferation in vitro

**FIGURE 2:**
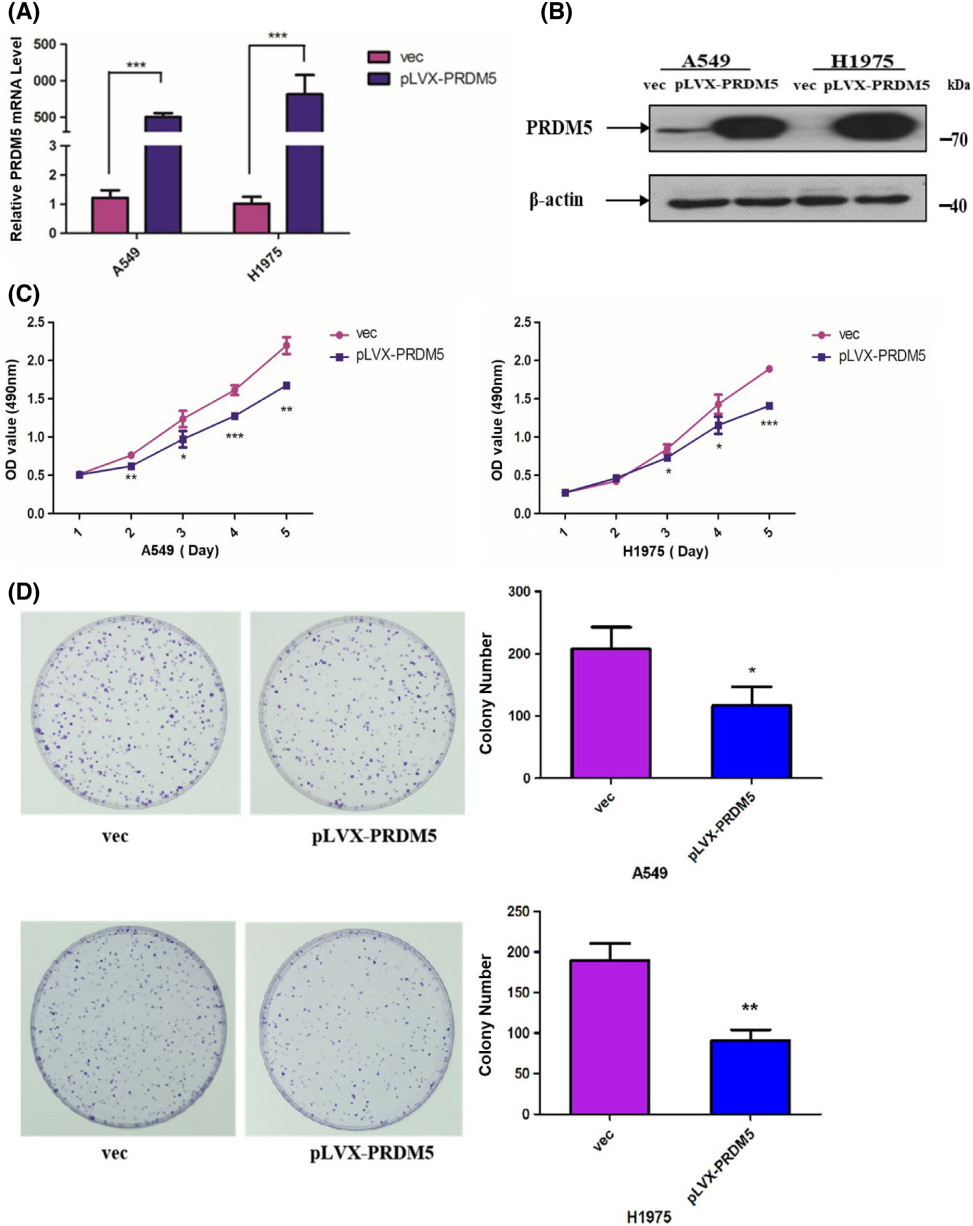
Upregulation of PRDM5 inhibits lung adenocarcinoma cell proliferation in vitro. (A) mRNA levels were assessed in PRDM5‐overexpressing lung adenocarcinoma cell lines by qPCR. (B) Protein levels were examined in PRDM5‐overexpressing lung adenocarcinoma cell lines by Western blotting. (C) MTT assay in A549 (left) and H1975 cells (right). (D) Colony formation assay in H1975 and A549 cells. *p < 0.05; **p < 0.01; ***p < 0.001.

To identify the effect of PRDM5 on cell proliferation, we established A549 and H1975 stable cell lines that overexpressed PRDM5 (pLVX‐PRDM5) or carried empty vector as controls. Exogenous PRDM5 expression was examined by qPCR and Western blotting, as shown in Figure 2A,B. Upregulation of PRDM5 suppressed cell growth in both A549 and H1975 cells in vitro (Figure 2C). Cell proliferation suppressed by PRDM5 was also further verified by a colony formation assay. As shown in Figure 2D, compared with the control group, the number of clones in PRDM5‐overexpressing A549 and H1975 cells was obviously lower. To elucidate whether PRDM5 upregulation affects the cell cycle, we performed a FACS assay in PRDM5‐overexpressing A549 cells. The results showed that PRDM5 had no correlation with the cell cycle in A549 cells (Figure S1).

### 3.3 Deregulation of PRDM5 promoted proliferation of lung adenocarcinoma cell lines in vitro

**FIGURE 3:**
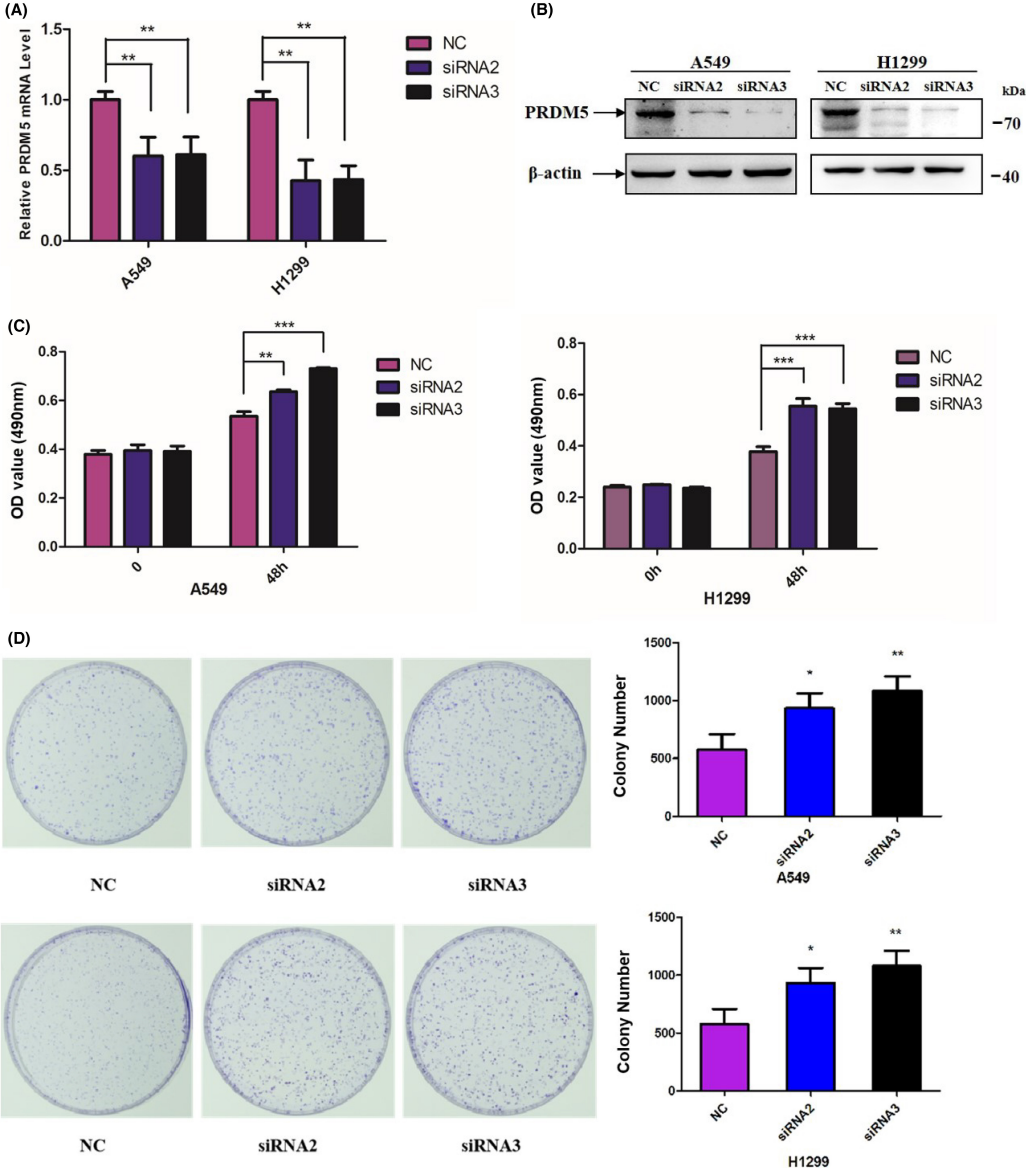
Reduced PRDM5 promotes cell proliferation. PRDM5 mRNA (A) and protein levels (B) were examined in H1299 and A549 cells with PRDM5‐siRNA or control. (C) MTT assays were performed 48 h posttransfection in A549 (left) and H1299 cells (right). (D) Colony formation assay of PRDM5 knockdown and control A549 (up) and H1299 cells (down). *p < 0.05, **p < 0.01.

To confirm the role of PRDM5 in cell proliferation, we knocked down PRDM5 by transient transfection of two siRNAs into A549 and H1299 cell lines. The mRNA and protein levels of PRDM5 expression were identified at 48 h posttransfection (Figure 3A,B). MTT and colony formation assays showed that PRDM5 knockdown (si‐PRDM5) markedly promoted cell proliferation compared with si‐Control in vitro (Figure 3C,D). Taken together, the results demonstrated that deregulation of PRDM5 gave rise to increased cell proliferation in lung adenocarcinoma cells.

### 3.4 PRDM5 suppressed orthotopic tumor growth in vivo

**FIGURE 4:**
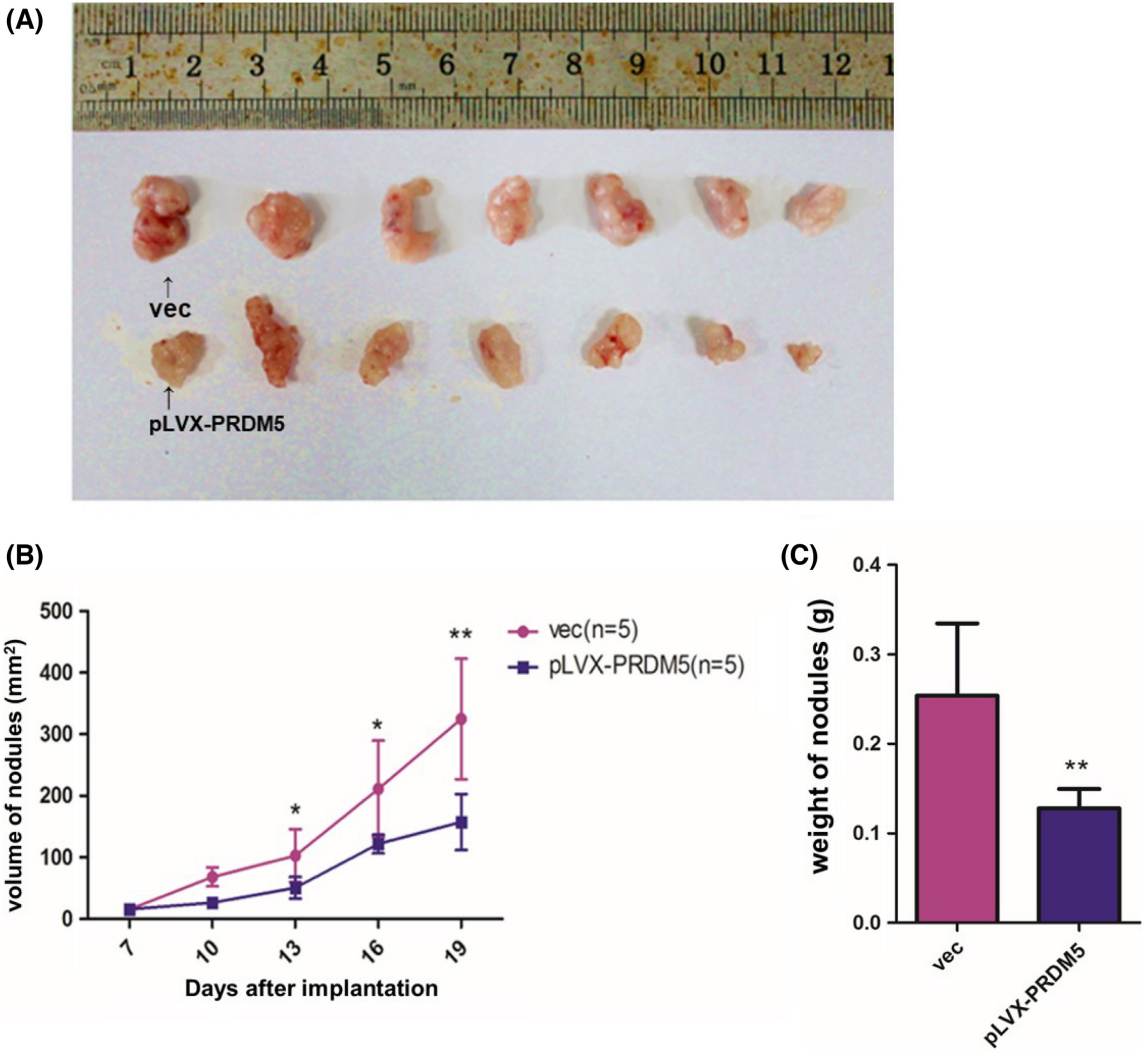
Overexpression of PRDM5 suppresses tumor growth in nude mice. Four‐week‐old female Balb/C nude mice (*n* = 14) were randomly divided into two groups. A549 cells stably overexpressing PRDM5 were orthotopically inoculated into the right shoulder of each mouse with 1 × 10^6^ cells. (A) Pictures of tumors were taken from the mice of each group 20 days after subcutaneous injections. (B) The tumor volume was measured every 3 days, and the results of two groups (PRDM5‐overexpressing and vector control) are presented as tumor growth curves. (C) tumor weight of the PRDM5‐overexpressing group versus the vector control groups. *p < 0.05, **p < 0.01.

To examine whether PRDM5 overexpression affected tumorigenesis in vivo, A549 cells stably overexpressing PRDM5 (1 × 10^6^ cells) were orthotopically inoculated into the right shoulder of each mouse. Tumor xenograft mice injected with PRDM5‐overexpressing A549 cells showed significantly reduced tumor formation ability (Figure 4A), tumor volume (Figure 4B) and tumor weight (Figure 4C) compared to control mice. Consistent with the results in vitro above, the overexpression of PRDM5 suppressed the tumorigenesis of LUAD in vivo.

### 3.5 PRDM5 enhanced the transcriptional activity of SOCS1

**FIGURE 5:**
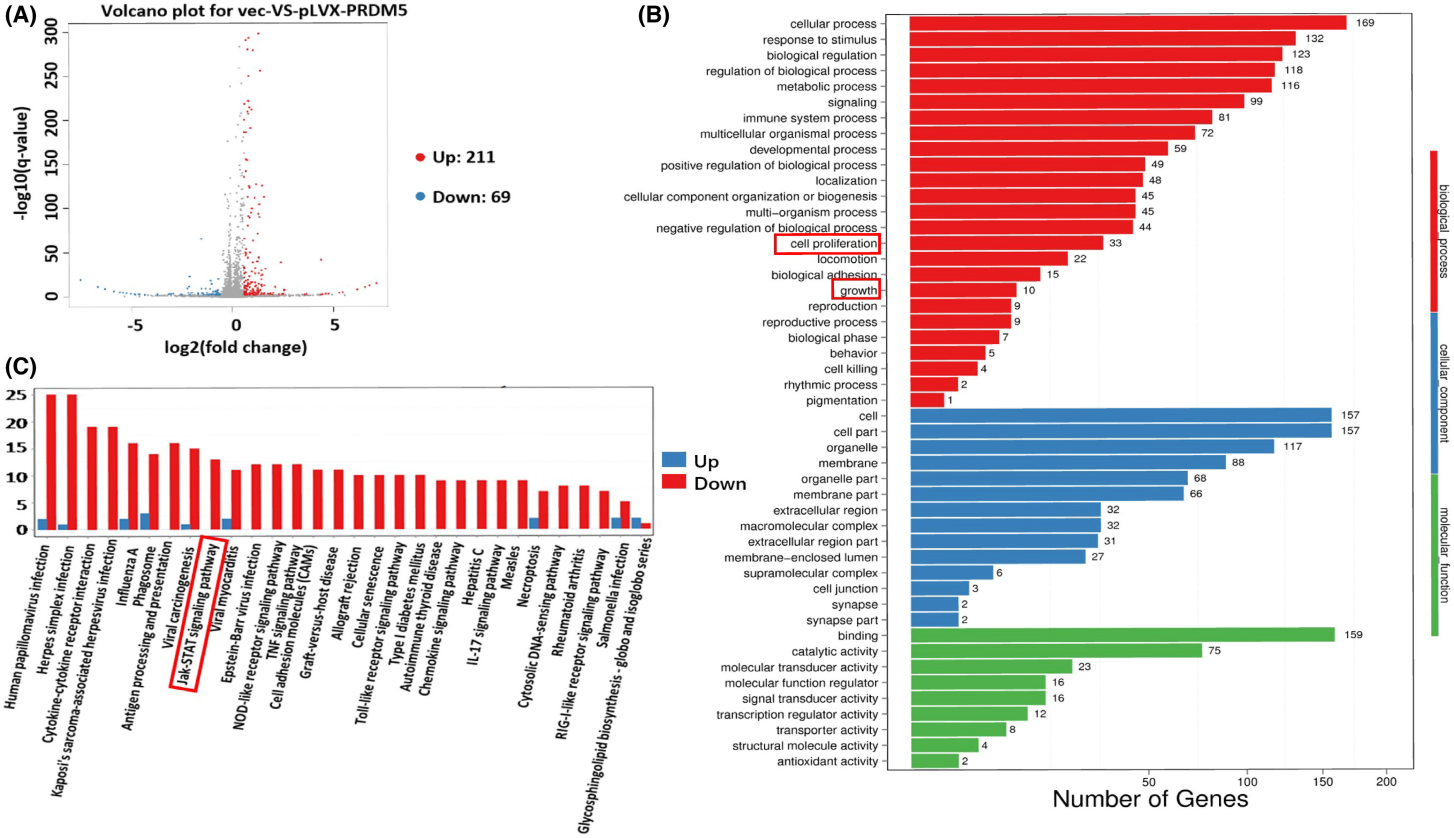
(A) RNA‐sequencing analysis reveals an altered transcriptome profile in PRDM5‐overexpressing A549 cells. Differentially expressed genes (*n* = 280) were selected between PRDM5‐overexpressing (pLVX‐PRDM5) and control (vec) A549 cells (*p* < 0.05, fold change = 1.5). (B) Gene ontology (GO) function classification map of differentially expressed genes (PRDM5‐overexpressing versus control A549 cells, ranked by *p* value). (C) KEGG pathway enrichment was determined by differentially expressed genes in PRDM5‐overexpressing A549 cells (*p* < 0.05 was considered significant).

To explore the potential genes that may be regulated by PRDM5, RNA‐sequencing analysis was performed to screen the target gene profiles in PRDM5‐overexpressing A549 cells. The database analysis revealed that among 280 differentially expressed transcripts identified, there were 211 upregulated genes and 69 downregulated genes (Figure 5A, *p < 0.05, fold change = 1.5). GO function analysis showed that cellular process ranked first, as well as cell growth and death in the KEGG pathway analysis (Figure 5B,C). According to the previous results of cell phenotypic experiments, we preliminarily screened some differentially expressed genes involved in proliferation and cell growth. We found that SOCS1 protein, a member of the suppressor of cytokine signaling (SOCS) family, was involved in cell growth and was an important component of the JAK/STAT pathway. Interestingly, enrichment of the JAK/STAT signaling pathway occurred in PRDM5‐overexpressing A549 cells (Figure 5C).

**FIGURE 6:**
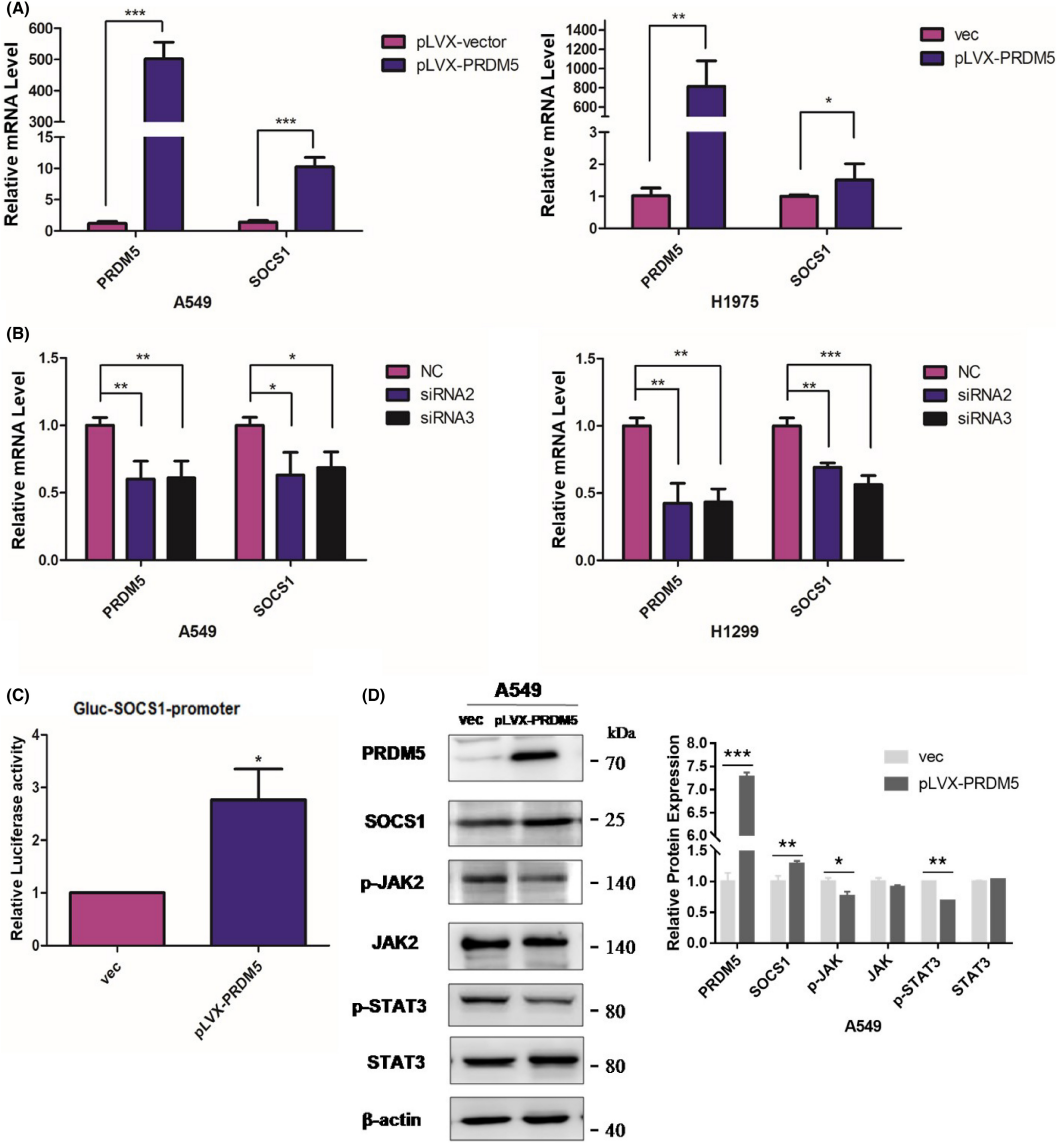
SOCS1 is positively regulated by PRDM5 and promotes cell proliferation by suppressing the JAK2/STAT3 pathway in lung adenocarcinoma cells. (A) PRDM5 and SOCS1 mRNA levels in PRDM5‐overexpressing A549 (left) and H1975 cells (right) were analyzed by qPCR. (B) PRDM5 and SOCS1 mRNA levels in PRDM5‐knockdown A549 (left) and H1299 cells (right). (C) Ectopic PRDM5 expression significantly increased SOCS1‐dependent Gluc activity. Mean ± SD from three individual experiments. (D) JAK2/STAT3 signaling pathway‐associated protein expression in PRDM5‐overexpressing and empty vector A549 cells was examined by Western blotting. *p < 0.05; **p < 0.01; ***p < 0.001.

To identify the results of RNA‐seq and determine whether PRDM5 may influence the expression of SOCS1, we first overexpressed PRDM5 in A549 and H1975 cell lines and knocked down PRDM5 in A549 and H1299 cells. The results showed that PRDM5 promoted SOCS1 expression at the mRNA level (Figure 6A,B). Then, the transcriptional activity of SOCS1 was examined in PRDM5‐overexpressing 293 T cells by the GLuc‐On‐promoter reporter assay. The results showed that PRDM5 significantly enhanced the transcriptional activity of SOCS1 (Figure 6C), implying that PRDM5 may suppress lung tumorigenesis by positively regulating SOCS1.

### 3.6 PRDM5 might suppress the JAK/STAT pathway by upregulating SOCS1 expression in lung adenocarcinoma

Although SOCS1 has been found to have growth‐suppression activity in human hepatocellular carcinoma, it is also recognized as a tumor promoter in cancers due to different cellular contexts. To investigate the potential mechanism by which PRDM5 deregulation promoted the proliferation of lung adenocarcinoma cells, we tested the expression levels of components of the JAK2/STAT3 pathway in A549 cells overexpressing PRDM5. In addition to SOCS1 upregulation, the expression of phospho‐JAK2 and phospho‐STAT3 in PRDM5‐overexpressing A549 cells was decreased compared with that in control cells (Figure 6D). The results above indicated that inhibition of the JAK2/STAT3 pathway triggered by SOCS1 might be mediated by PRDM5 in lung adenocarcinoma cells.

## 4 DISCUSSION

As a transcriptional regulator, PRDM5 has been implicated as a tumor suppressor and to be silenced in several cancers.^8^, ^15^, ^16^ Similarly, we observed the downregulation of PRDM5 expression and its association with a poorer survival rate in LUAD patients. Moreover, overexpression of PRDM5 suppressed both orthotopic tumor growth in vivo and cell proliferation in vitro, indicating its tumor suppressive property in lung adenocarcinoma.

Mechanically, numbers of previous studies focused on the detection of *PRDM5* gene methylation status,^7^, ^8^, ^9^, ^17^ which was determined contributing to the silencing of PRDM5 in several tumor tissues include lung cancer, few literatures elucidated the potential mechanisms of PRDM5 tumor suppressive functions in LUAD carcinogenesis. As an epigenetic regulator and a transcription factor, PRDM5 should exert its tumor suppressive functions through transcription and cell signaling. Despite the finding that the suppression of WNT/β‐catenin signaling on account of ectopic PRDM5 expression was determined in limited cancer cell lines,^12^ we first explored and identified the inhibition of JAK2/STAT3 pathway in LUAD cell lines with PRDM5 overexpression. In addition, the promoter activity of SOCS1 was positively regulated by PRDM5, implying the possible mechanism underlying the tumor suppressive activities of PRDM5 in LUAD cell proliferation.

As a member of the suppressor of cytokine signaling (SOCS) family, SOCS1 has been recognized crucial for regulating JAK–STAT pathway both in cell proliferation and neoplastic transformation.^18^, ^19^ Meanwhile, silencing of SOCS1 has also been found in 75% of melanomas,^20^ more than 50% of primary tumors of hepatocellular carcinoma^21^ and 44% of gastric carcinomas.^22^ Therefore, evaluating SOCS1 expression and its correlated functions may have prognostic significance in cancers.^23^, ^24^ Our analysis and identification based on RNA‐seq results suggested that the transcriptional activity of SOCS1 can be upregulated by PRDM5 in A549 cells. PRDM5 repression in lung adenocarcinoma cell lines suppressed the expression of SOCS1, thereby regulating downstream signaling pathways or target proteins.

Hyperactivation of the JAK–STAT pathway in many tumor malignancies has aroused concern and has become a target of cancer drug development.^25^ Toward identifying the enrichment of the JAK/STAT signaling pathway based on our KEGG pathway analysis, we determined the decreased phosphorylation of JAK2 and STAT3 in PRDM5‐overexpressing A549 cells, which was in accord with the previous results in NSCLC patients.^20^ Dysregulation of JAK/STAT mediated by SOCS1 has been demonstrated mainly in cytokine signaling,^11^, ^18^, ^21^, ^26^, ^27^ indicating that the cell proliferation control dependent on SOCS1 has a similar mechanism in the suppression of cancer growth. According to our results, overexpressed PRDM5 is likely to upregulate the promoter activity of SOCS1 and further suppress the JAK2/STAT3 pathway by inhibiting the phosphorylation of JAK2 and STAT3, thus repressing cell proliferation in lung adenocarcinoma.

Although previous study showed that overexpression of PRDM5 led to G2/M arrest and apoptosis of tumor cells,^8^ we failed to acquire consistent results in lung adenocarcinoma cell lines. On the basis of our results, PRDM5 may exert its tumor suppressive function by regulating cell proliferation rather than the cell cycle in LUAD. Toward determining the effect of SOCS1 expression on the cell proliferation of LUAD and its association with PRDM5, more in vitro and in vivo evidence will be needed to uncover the role of PRDM5 in lung carcinogenesis.

In summary, our study suggests that PRDM5 functions as a tumor suppressor in lung adenocarcinoma. Ectopic PRDM5 expression promotes the transcriptional activity of SOCS1 and deregulates the JAK2/STAT3 signaling pathway. The findings will provide significant clues for the targeted treatment of lung adenocarcinoma in the clinic.

## AUTHOR CONTRIBUTIONS

Y.R and Y.W established the initial topic and conducted the crucial part of this study. Y.W, L.F, M.M, and L.G accomplished the research work. Y.R, Y.W, and X.L took part in writing the manuscript. The study was supervised by J.Y. L.X, C.S, and J.H had given the significant comment to this work.

## FUNDING INFORMATION

This study was supported by grants 82070008 (to L.G.) and 32070724 (to J.Y.) from the National Natural Science Foundation. Grant 2019KJ171 (to Y. R.) from Scientific Research Project of Tianjin Education Commission (Natural Science).

## CONFLICT OF INTEREST

All the authors disclose that they have no conflict of interests.

## ETHICAL APPROVAL STATEMENT

The mice used in this study had been approved by the Institutional Animal Care and Use Committee, Tianjin Medical University.

## Supporting information


Figure S1

## Data Availability

All the data were included in this paper for the evaluation. Additional relevant data might be available in consulting with the authors.
