# Supplementary figures and images for: Deregulation of PRDM5 promotes cell proliferation by regulating JAK/STAT signaling pathway through SOCS1 in human lung adenocarcinoma
Source: Cancer Med. 2022 Sep 20;12(4):4568–78. doi: 10.1002/cam4.5251 (PMC9972168; doi:10.1002/cam4.5251)

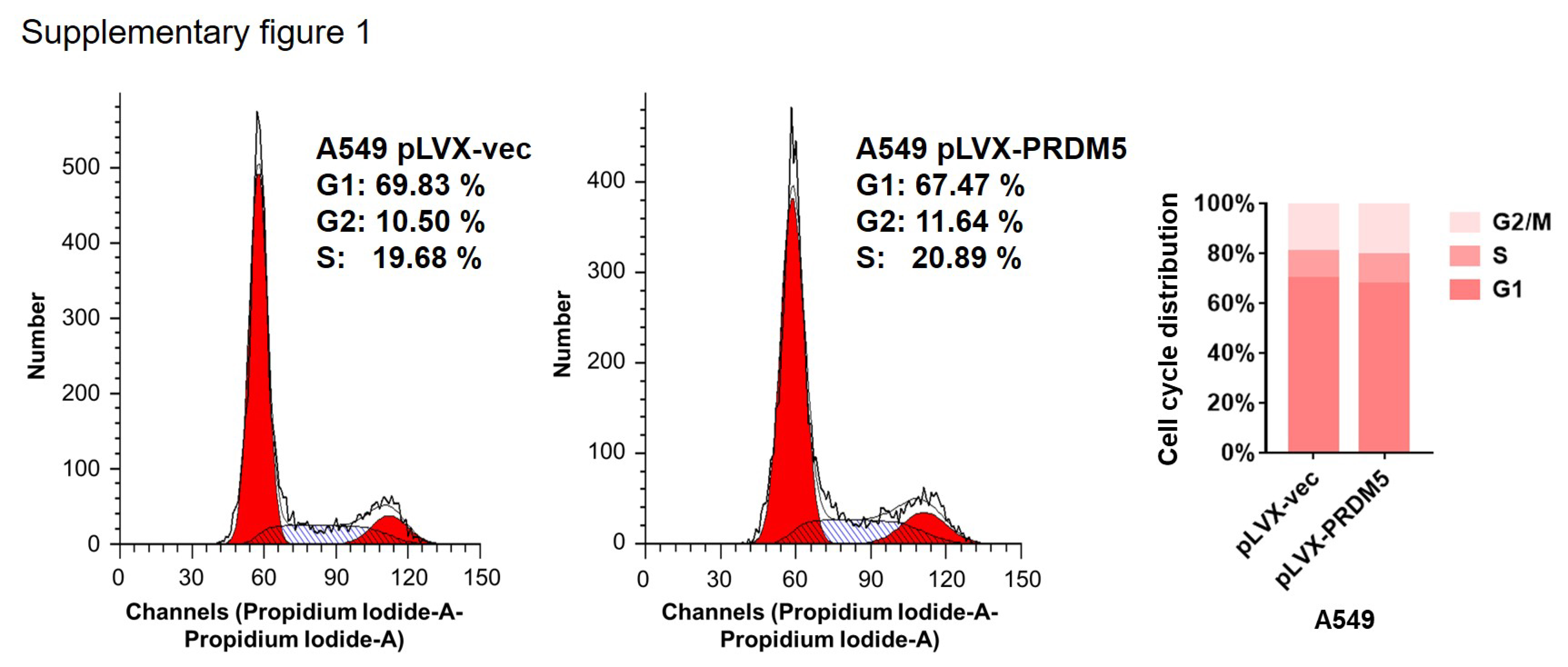

Supplement: Supplementary file 1 — Figure S1 [file CAM4-12-4568-s001.jpg]
